# Inpatient medical costs and their composition among patients with stomach cancer who underwent surgery by tumor location: a hospital-based retrospective study in China

**DOI:** 10.1186/s12962-026-00837-7

**Published:** 2026-10-01

**Authors:** Zeng-Bao Hu, Shang-Qi Chen, Stuart McDonald, Meng-Meng Chen, Hua Yu, Yi Lin

**Affiliations:** 1 School of Accounting and Finance, Yantai Institute of Technology, Yantai, Shandong Province China; 2 Department of Gastrointestinal and Hernia Ward, Ningbo No. 2 Hospital, Wenzhou Medical University, No. 41 Xibei Street, Ningbo, Zhejiang Province China; 3 Faculty of Humanities and Social Sciences, University of Nottingham Ningbo China, No.199 Taikang East Road, Ningbo, Zhejiang Province China; 4 Department of Clinical Nutrition, Ningbo No. 2 Hospital, Wenzhou Medical University, No. 41 Xibei Street, Ningbo, Zhejiang Province China

**Keywords:** China, Stomach cancer, Medical cost, Economic burden, Stage at diagnosis, Tumor site, Resource allocation

## Abstract

**Background:**

Stomach cancer (SC), the third leading cause of cancer-related death in China, poses a considerable challenge to the Chinese healthcare system. Evidence on inpatient medical costs and cost composition among patients with SC who underwent surgery in China remains limited. Using hospitalization billing data from Ningbo, China, this study estimates the total medical costs and cost composition of SC to gain insights into the economic burden due to SC in China.

**Methods:**

A hospital-based retrospective observational study was conducted using data from a tertiary hospital in Ningbo, China. SC-related medical expenditure and the sociodemographic and clinicopathologic characteristics of inpatients were obtained from the hospital billing system and inpatient medical records. Patients aged ≥ 18 years, with a primarily diagnosis of SC who underwent surgery between 2020 and 2023 were included in the study. The proportional breakdown of SC medical expenditure was analyzed by separating the expenditure into five major categories: materials, medications, surgery, examinations, and hospital services. All expenditure data were converted to 2020 Chinese Yuan.

**Results:**

A total of 1,102 inpatients were included, where 69.51% were male, 69.06% were at stages I and II, and 58.89% had tumors in the lower part of the stomach. Inpatient medical costs were generally higher among older patients and patients with more advanced tumor stage. Materials, medications, and examinations were the major cost drivers of SC medical costs, jointly accounting for 73–74% of total costs across tumor sites.

**Conclusions:**

Medical costs among SC inpatients were substantial and varied across patients’ sociodemographic and clinicopathological characteristics, particularly by age, tumor stage, and tumor site. These findings provide contemporary cost estimates that may inform hospital budgeting, procurement of medical materials and medications, and the refinement of reimbursement arrangements for SC hospitalization. They may also provide insights for future economic evaluations of SC prevention and treatment strategies.

**Supplementary Information:**

The online version contains supplementary material available at https://doi.org/10.1186/s12962-026-00837-7.

## Introduction

Stomach cancer (SC) is the fifth most common cancer worldwide, with 0.97 million new cases and 0.66 million new deaths occurring in 2022 [1]. Although SC incidence and mortality rates have steadily declined in most countries over the past decades, the absolute number of cases and deaths is still expected to increase in the future due to a growing aging population [2]. In particular, SC is one of the most common public health problems in eastern Asia, Eastern Europe, and South America, where the incidence and mortality rates are higher than in other regions [1]. In 2022, 0.36 million new cases and 0.26 million deaths occurred in China, making SC the fifth most commonly diagnosed cancer and the third leading cause of cancer-related death in China [3]. The eastern region has the second highest incidence and mortality rates of SC in China, with the age-standardized incidence rate and mortality rate estimated at 21.9 and 14.8 per 100,000 in 2016, respectively [4]. Ningbo is a major coastal city in eastern China with an aging population and a substantial demand for SC care, making it a relevant setting for examining contemporary inpatient medical costs.

SC can impose a substantial economic burden through direct medical costs, productivity losses, and other indirect costs. From a clinical perspective, SC is often detected at a later stage because of the lack of visible signs. Its cause is largely attributed to unhealthy lifestyles and the Helicobacter pylori (H. pylori) infection, which are common in many countries, imposing a significant economic burden on patients and the society [5].It is estimated that the total economic costs of SC from 2020 to 2050 will be $1,449.00 billion globally, representing 3.20% of global GDP during this period [6]. The medical costs of SC were $190.20 billion in the United States in 2015 [7] and €5.00 billion in Europe in 2018 [8]. A previous multicenter study in China reported direct expenditures of approximately $9,899 per patient between 2012 and 2014 and found that this was unmanageable for nearly 80% of affected households [9].

China implemented the Diagnosis-Related Group (DRG) payment system reform in 2012, aiming to reduce the economic burden of diseases, particularly for cancers, by reducing unnecessary medical expenditures for inpatients and optimizing medical resource utilization [10, 11]. Against this policy background, contemporary evidence on SC-related medical costs and their composition is needed to support hospital resource planning, payment policy, and future economic evaluations of SC care. However, although numerous studies have focused on the total medical costs of SC, little information is available on the compositional categories of SC medical costs and their proportions in the total costs in China [9–13]. In addition, the literature on SC medical costs in the 2020s is limited. Two multicenter studies reported the proportional breakdown of SC medical costs based on data from the 2000s and 2010s [14, 15], which may not reflect the situation in the 2020s given the changes in treatment strategies and medical policies over the past decade. Examining SC cost composition across years in the 2020s may also provide further insight into whether recent cost patterns have remained stable or changed in the context of evolving treatment practices and healthcare policies.

Previous studies have reported differences among SC patients with different tumor locations in clinicopathological characteristics, surgical procedures, and treatment patterns [16, 17]. These differences may lead to variation in healthcare resource use and may also affect how patient characteristics, such as sex, age, and tumor stage, are associated with medical costs. Therefore, examining these associations separately by tumor site may provide more detailed information on cost patterns among different tumor-site groups that could help improve hospital resource planning and allocation. In addition, previous literature on SC costs has generally treated SC as a single category, providing limited information on whether the composition of medical costs and the factors associated with these costs vary across tumor sites [9, 13–15].

Therefore, this study estimates total inpatient medical costs and their composition among patients with SC who underwent surgery and to explore whether the associations between patient characteristics and these costs showed different patterns across tumor sites. Using recent hospital billing data from a tertiary hospital in Ningbo, China, patients were stratified by tumor site, and the associations of age, sex, and TNM stage with total and category-specific medical costs were examined separately within each tumor site group.

## Methodology and data

### Study design and patients

We conducted a hospital-based retrospective observational study using patient-level data from a tertiary hospital in Ningbo, Zhejiang Province, China. This study was approved by the Ethics Committee of the Ningbo No.2 Hospital (No. PJ-NBEY-KY-2025-033-01). The following inclusion criteria were used for selecting study subjects:

1)The patient was newly diagnosed with SC, subsequently hospitalized, and underwent surgery between January 1, 2020, and December 31, 2023;

2)The patient was aged ≥ 18 years at the time of initial diagnosis;

3)Information on age, sex, tumor site, cancer stage, and SC-related medical costs during hospitalization was available for each patient.

**Fig. 1:**
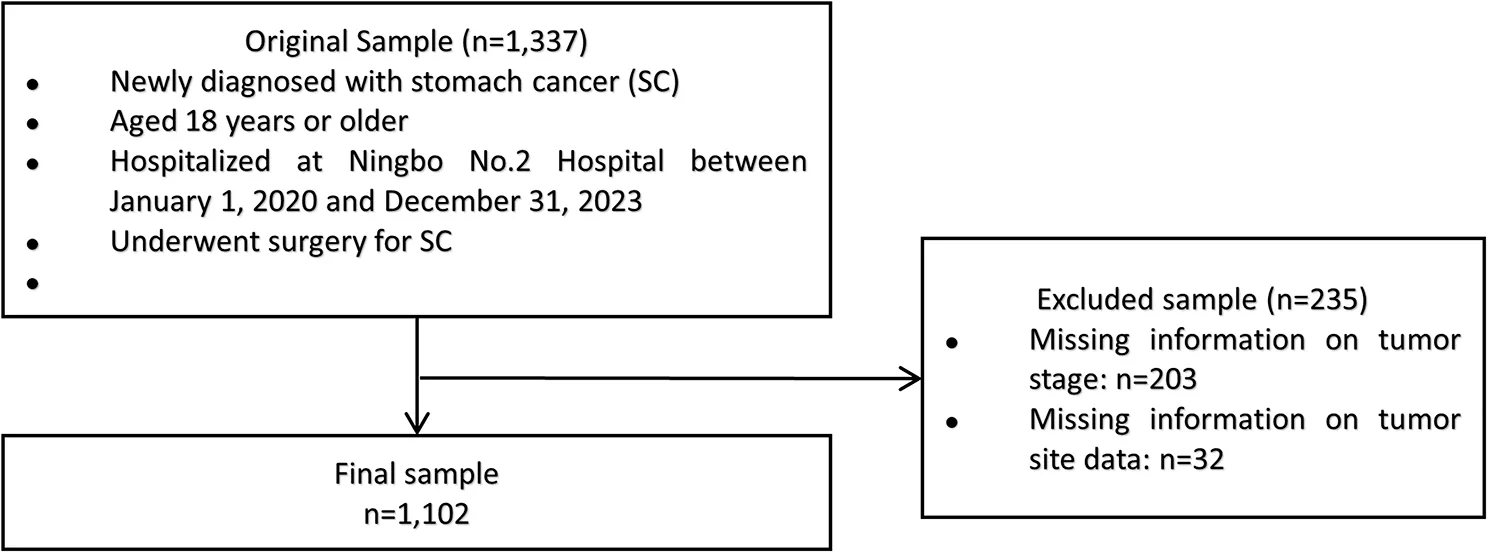
Flowchart of Patient Selection

The patient selection process is illustrated in Fig. 1. A total of 1,337 inpatients were diagnosed with SC between 2020 and 2023, and 1,102 were included in the present study, with 203 and 32 inpatients excluded due to the lack of information on tumor stage and tumor site, respectively. All included patients were newly diagnosed with SC and were admitted to the study hospital for their first hospitalization following diagnosis. No patient had more than one hospitalization at the study hospital during the study period.

The present study specifically focuses on patients with SC who underwent surgery to generate a clinically representative patient group for analyzing the SC-related medical costs during hospitalization, because surgical treatment is the key therapy option for SC treatment that requires substantial and well-defined use of medical resources. Patients were categorized into four cancer stage groups (I, II, III, IV), based on the American Joint Committee on Cancer (AJCC) TNM system [18]. Age at diagnosis was defined as the patient’s age at the initial diagnosis of SC. Because all included patients were newly diagnosed during the study period and admitted to the study hospital for their first hospitalization following diagnosis, age at diagnosis effectively corresponded to age at hospital admission, with no meaningful interval between the initial diagnosis and the hospitalization analyzed. Based on the age at diagnosis, patients were divided into three age groups: <60, 60–79, and > 79 years. Patients were separated into four groups based on tumor sites and analyzed separately: the upper part of the stomach, including the cardia and fundus; the middle part, corresponding to the body; the lower part, including the antrum and pylorus; and multicentric SC, defined as two or more distinct tumors within the stomach [19].

### Definition and measurement of inpatient medical costs

Medical cost data were obtained from the hospital billing system and represented the total amount of inpatient medical expenditure recorded before reimbursement by public health insurance or third-party payers. For each patient, the cost data corresponded to their first hospitalization at the study hospital following their diagnosis of SC. All included patients had only one hospitalization at the study hospital during the study period. Therefore, the reported costs represent expenditure for a single hospitalization episode rather than cumulative costs across multiple admissions. Total inpatient medical costs were divided into five categories: material costs, medication costs, surgery costs, examination costs, and hospital service costs. Material costs included costs of disposable medical supplies and surgical materials used during hospitalization, such as bandages, syringes, needles, and surgical instruments. Medication costs included costs of medications administered during hospitalization, including both prescription drugs and over-the-counter (OTC) drugs administered in different ways, such as orally, by infusion, or as drops. Surgery costs included the surgeon’s fees, the anesthesia fees, and the facility fees. Examination costs included the costs on all medical examinations, including physical examinations, endoscopy examinations, imaging examinations, and laboratory tests. Hospital service costs included costs of consultation, nursing services, rehabilitation, hospital accommodation, and blood transfusion. To facilitate comparisons across years, all costs were converted to 2020 Chinese yuan using the annual Consumer Price Index (CPI) for China.

### Statistical analysis

This study described and analyzed the pooled data of SC-related medical costs and sociodemographic and clinicopathologic characteristics of inpatients. This study separately estimated the SC-related medical costs for patients with tumors located in the lower, middle, and upper parts of the stomach, dividing them into five major categories (material costs, medication costs, surgery costs, examination costs, and hospital service costs). Because cost distributions were non-normal, SC-related medical costs were summarized using medians and interquartile ranges 25–75%. Categorical variables, including sex, age group, and cancer stage were presented as numbers with percentages. The composition of medical costs was also descriptively summarized by year to describe temporal patterns in cost composition during the study period of 2020–2023.

When comparing costs across groups, the analytical strategy prioritized distribution-aware testing. The test used for each comparison was selected based on the data distribution to ensure the robustness and appropriateness of the testing. Specifically, the Shapiro-Wilk test and visual inspection of histograms indicated that cost data were non-normally distributed. The Jonckheere-Terpstra test was used to compare costs between ordered groups (e.g., age groups, TNM stages), whereas the Kruskal-Wallis test was used for unordered groups (e.g., sex, tumor sites). Categorical variables were compared using the chi-square test.

Medical cost outcomes were logarithmically transformed before regression. The multivariable Generalized Linear Models (GLMs) with a Gaussian distribution and an identity link function were used to examine the associations between SC costs and patient characteristics (sex, age at diagnosis, TNM stage), stratified by tumor site (upper part of stomach, middle part of stomach, lower part of stomach, and multicentric SC). The Gaussian family with identity link was selected for log-transformed cost outcomes because logarithmic transformation reduced the skewness of the original cost distributions. Heteroskedasticity-robust standard errors were used to handle the heteroskedasticity problem. Calendar years were included to control for potential time trends. Variance inflation factors were calculated to assess the existence of potential multicollinearity problem.

Three additional analyses were conducted. First, as a sensitivity analysis, all cost data were winsorized at the 1st and 99th percentiles, and the multivariable regression analyses were repeated to assess whether the findings were influenced by extreme cost values. Second, age was further categorized into < 60, 60–64, 65–69, 70–74, 75–79, and > 79 years, and the regression analyses were repeated to examine whether the findings were sensitive to a more granular age classification. Third, pooled multivariable regressions were conducted with tumor site as the key independent variable while adjusting for the same covariates included in the main regression analyses to examine associations between tumor site and SC-related medical costs. For all the three additional analyses, GLMs with a Gaussian distribution and an identity link function were used.

All statistical tests were two-sided, and a p-value less than 0.05 was considered statistically significant. Statistical analyses were performed using STATA 17.0 (2021).

## Results

A total of 1,102 patients were included for analysis in the analysis, and the majority of the patients were male (69.51%) and aged between 60 and 79 years (74.95%) (Table 1). Patients at TNM stages I, II, III, and IV accounted for 46.28%, 22.78%, 26.95%, and 3.99% of the total sample, respectively. The most common tumor site was the lower part of the stomach (58.89%), followed by the middle part (28.13%) and the upper part (7.26%), while only 5.72% had multicentric SC. The male proportions in patients with tumors in the upper part of the stomach (86.25%) were higher than those in patients with tumors in other parts of the stomach (*p* = 0.005). There were significant differences in cancer stage across tumor sites (*p* < 0.001), as multicentric SC had a higher proportion of stage II patients, whereas middle stomach cancer had a higher proportion of stage III patients.

**Table 1:** Characteristics and medical costs of patients with stomach cancer who underwent surgery by tumor site

Tumor Site Characteristics	Total Sample	Upper Part of Stomach	Middle Part of Stomach	Lower Part of Stomach	Multicentric Stomach Cancer	*P* ^a^
N (%)						
**Number of Patients**	1,102 (100.00%)	80 (7.26%)	310 (28.13%)	649 (58.89%)	63 (5.72%)	
**Sex**						0.005
Male	766 (69.51%)	69 (86.25%)	211 (68.06%)	439 (67.64%)	47 (74.60)	
Female	336 (30.49%)	11 (13.75%)	99 (31.94%)	210 (32.36%)	16 (25.40)	
**Age at Diagnosis**						0.348
≤ 59	169 (15.34%)	10 (12.50%)	59 (19.03%)	93 (14.33%)	7 (11.11%)	
60–79	826 (74.95%)	60 (75.00%)	219 (70.65%)	496 (76.43%)	51 (80.95%)	
≥ 80	107 (9.71%)	10 (12.50%)	32 (10.32%)	60 (9.24%)	5 (7.94%)	
**Cancer Stage**						< 0.001
TNM I	510 (46.28%)	40 (50.00%)	127 (40.97%)	323 (49.77%)	20 (31.75%)	
TNM II	251 (22.78%)	16 (20.00%)	68 (21.94%)	141 (21.73%)	26 (41.27%)	
TNM III	297 (26.95%)	20 (25.00%)	101 (32.58%)	165 (25.42%)	11 (17.46%)	
TNM IV	44 (3.99%)	4 (5.00%)	14 (4.52%)	20 (3.08%)	6 (9.52%)	
Median [interquartile range 25–75%]						
**Total Cost**	47,539.68	47,719.18	48,798.06	47,138.75	49,550.05	0.041
	[42,648.20–54,883.05]	[42,124.3–55,713.89]	[43,266.73-56,785.00]	[42,151.60–53,753.64]	[44,778.00–55,922.25]	
**Material Cost**	17,834.57	18,065.46	18,539.50	17,521.00	18,025.22	0.255
	[14,619.05-21,352.82]	[14,308.46-21,916.50]	[14,945.58-21,753.00]	[14,588.92-21,099.11]	[13,730.36-21,407.33]	
**Medication Cost**	9,416.06	9,378.79	9,697.36	9,225.46	10,191.28	0.142
	[7,805.75-11,784.26]	[8,192.50–11,573.48]	[7,894.07-12,276.00]	[7,717.00–11,682.85]	[8,542.19-11,712.34]	
**Surgery Cost**	7,575.06	7,771.14	7,584.80	7,540.33	7,663.43	0.095
	[7,000.00–8,236.66]	[7,139.46-8,355.44]	[7,012.88-8,379.24]	[6,949.47-8,138.70]	[7,133.00–8,396.50]	
**Examination Cost**	7,696.32	7,952.86	7,798.10	7,607.53	7,631.43	0.104
	[6,776.48-9,478.69]	[6,709.76-9,687.73]	[6,916.59-10,003.89]	[6,700.29-9,258.67]	[6,760.93-10,770.65]	
**Hospital Service Cost**	4,426.40	4,458.51	4,532.05	4,397.42	4,407.37	0.348
	[3,616.13-6,030.72]	[3,697.28-5,783.14]	[3,672.00–6,276.43]	[3,539.28-5,750.24]	[3,716.23-6,565.91]	

Note: Values are expressed as frequency (%), or as median [interquartile range 25–75%]

Costs are measured by 2020 CNY (¥)

^a^ Comparisons are made between different tumor site groups, using the Chi-2 Test for categorical variables and the Kruskal-Wallis Test for continuous variables, respectively. Results are presented as P-values

The median treatment cost per patient was ¥47,539.68. The median costs per patient for materials, medications, surgery, examinations, and hospital services were ¥17,834.57, ¥9,416.06, ¥7,575.06, ¥7,696.32, and ¥4,426.40, respectively (Table 1). Table 2 displays changes in the composition of SC-related medical costs from 2020 to 2023. Except for the multicentric SC group, all tumor site groups exhibited an overall pattern of decreasing proportions of material and medication costs and increasing proportions of other cost categories. For patients with multicentric SC, the proportion of hospital service costs decreased, while the proportions of other cost categories increased or remained relatively stable.

**Table 2:** Composition of medical costs for stomach cancer from 2020 to 2023

Cost Composition	Year				Annual Growth Rate
	2020	2021	2022	2023	
Total Sample					
Material Cost	36.40%	37.02%	37.52%	33.40%	-2.65%
Medication Cost	21.18%	21.22%	21.55%	19.85%	-2.05%
Surgery Cost	14.79%	14.53%	15.51%	17.07%	5.02%
Examination Cost	16.38%	16.78%	16.29%	18.03%	3.38%
Hospital Service Cost	11.25%	10.46%	9.13%	11.66%	2.66%
**Upper Part of Stomach**					
Material Cost	35.14%	36.44%	37.72%	34.61%	-0.34%
Medication Cost	20.40%	19.95%	21.49%	18.46%	-2.87%
Surgery Cost	15.68%	15.48%	15.94%	17.17%	3.14%
Examination Cost	17.42%	17.23%	16.38%	18.18%	1.65%
Hospital Service Cost	11.36%	10.90%	8.48%	11.59%	3.49%
**Middle Part of Stomach**					
Material Cost	37.78%	35.17%	38.10%	34.11%	-3.01%
Medication Cost	20.75%	22.47%	21.41%	18.85%	-2.80%
Surgery Cost	14.72%	14.16%	15.23%	17.23%	5.64%
Examination Cost	16.38%	17.32%	16.16%	17.83%	3.12%
Hospital Service Cost	10.37%	10.87%	9.09%	11.98%	6.74%
**Lower Part of Stomach**					
Material Cost	36.18%	37.87%	37.38%	32.88%	-2.89%
Medication Cost	21.43%	20.87%	21.50%	20.25%	-1.80%
Surgery Cost	14.89%	14.60%	15.59%	17.07%	4.78%
Examination Cost	16.23%	16.41%	16.42%	18.09%	3.79%
Hospital Service Cost	11.28%	10.27%	9.10%	11.71%	2.76%
**Multicentric Stomach Cancer**					
Material Cost	31.63%	40.08%	35.92%	33.81%	3.49%
Medication Cost	22.40%	18.00%	22.69%	21.40%	0.23%
Surgery Cost	12.78%	15.21%	15.45%	16.43%	8.97%
Examination Cost	16.68%	17.18%	15.47%	17.98%	3.09%
Hospital Service Cost	16.51%	9.53%	10.47%	10.39%	-11.06%

Note: The average annual growth rate was calculated as the mean of the year-on-year percentage changes from 2020 to 2023

Table 3 displays the median cost by patient demographic characteristics for different tumor sites. SC medical costs generally increased with age and cancer stage levels, particularly among patients with tumors in the middle and lower parts of the stomach. Medication costs (*p* = 0.010) and examination costs (*p* = 0.022) were significantly higher for male patients with middle stomach tumors, whereas no significant sex difference in medical costs was observed for other tumor sites.

**Table 3:** Costs of stomach cancer by cost composition and demographic characteristics

Cost Composition Characteristics	Total Cost	Material Cost	Medication Cost	Surgery Cost	Examination Cost	Hospital Service Cost
**Upper Part of Stomach**						
**Sex** ^**a**^						
Male	48,431.49	18,128.00	9,345.30	7,749.27	7,833.50	4,500.49
Female	44,894.28	18,002.92	9,643.34	7,863.24	8,152.58	3,884.04
P-value	0.489	0.894	0.543	0.553	0.906	0.325
**Age at Diagnosis** ^**b**^						
≤ 59	49,508.28	18,822.16	9,494.54	8,067.33	7,587.55	3,961.37
60–79	45,724.37	18,065.46	9,332.65	7,718.43	7,850.79	4,381.07
≥ 80	51,096.35	15,542.16	11,360.54	8,028.56	9,576.29	6,825.69
P-value	0.636	0.132	0.317	0.455	0.093	0.003
**Cancer Stage** ^**b**^						
TNM I	49,144.85	19,817.24	9,329.15	7,771.14	7,846.65	4,494.18
TNM II	43,523.51	16,886.52	9,322.54	7,372.72	7,987.41	3,866.78
TNM III	47,985.42	14,894.87	10,149.51	7,784.74	8,453.84	4,932.44
TNM IV	42,983.03	15,993.69	7,697.38	7,844.33	6,667.31	4,553.83
P-value	0.085	0.013	0.551	0.362	0.672	0.935
**Middle Part of Stomach**						
**Sex** ^**a**^						
Male	49,085.52	18,445.09	9,876.58	7,584.87	8,145.69	4,655.67
Female	47,691.00	19,127.85	9,200.20	7,582.93	7,533.20	4,349.85
P-value	0.130	0.855	0.010	0.782	0.022	0.385
**Age at Diagnosis** ^**b**^						
≤ 59	48,754.13	20,015.00	9,200.20	7,599.61	7,324.59	4,258.50
60–79	47,691.00	17,752.67	9,593.60	7,483.97	7,954.41	4,519.88
≥ 80	55,694.76	19,464.80	11,539.81	8,081.27	10,435.00	5,177.94
P-value	0.030	0.203	< 0.001	0.327	< 0.001	0.009
**Cancer Stage** ^**b**^						
TNM I	46,778.86	19,114.45	9,161.00	7,542.27	7,431.49	4,077.00
TNM II	50,301.75	17,937.78	10,296.18	7,568.85	8,533.78	4,737.11
TNM III	49,194.25	18,985.00	9,876.58	7,552.00	7,903.87	4,758.99
TNM IV	57,628.24	17,994.18	12,425.88	8,755.58	10,459.02	6,512.06
P-value	0.007	0.640	0.007	0.069	< 0.001	< 0.001
**Lower Part of Stomach**						
**Sex** ^**a**^						
Male	47,338.51	17,835.00	9,379.24	7,558.00	7,588.92	4,358.77
Female	46,337.85	16,901.88	8,995.90	7,526.24	7,642.22	4,495.82
P-value	0.052	0.058	0.070	0.564	0.412	0.623
**Age at Diagnosis** ^**b**^						
≤ 59	46,078.00	18,511.18	8,585.00	7,488.85	7,157.44	3,820.21
60–79	46,979.69	17,508.32	9,236.64	7,514.08	7,611.09	4,369.13
≥ 80	49,923.89	16,538.46	10,443.11	7,837.49	9,841.17	5,718.18
P-value	< 0.001	0.158	< 0.001	0.023	< 0.001	< 0.001
**Cancer Stage** ^**b**^						
TNM I	45,326.87	16,510.00	8,741.03	7,515.03	7,266.60	4,146.68
TNM II	47,820.21	18,833.50	9,583.09	7,584.74	7,819.24	4,370.66
TNM III	48,429.00	17,816.33	9,522.30	7,513.12	8,353.74	4,788.00
TNM IV	47,928.88	16,384.50	10,296.60	7,996.43	7,920.17	6,043.49
P-value	< 0.001	0.010	< 0.001	0.209	< 0.001	< 0.001
**Multicentric Stomach Cancer**						
**Sex** ^**a**^						
Male	49,816.65	17,366.38	10,421.92	7,647.92	8,055.29	4,500.49
Female	49,295.34	20,474.88	9,236.18	7,792.77	7,435.79	4,033.49
P-value	0.752	0.312	0.185	0.681	0.636	0.528
**Age at Diagnosis** ^**b**^						
≤ 59	46,131.91	19,406.22	8,815.71	6,964.11	7,192.42	3,589.72
60–79	49,048.56	18,902.00	9,943.51	7,663.43	7,631.43	4,407.37
≥ 80	55,593.66	13,938.00	13,293.36	8,711.93	14,102.08	7,242.48
P-value	0.080	0.124	0.189	0.019	0.003	0.001
**Cancer Stage** ^**b**^						
TNM I	47,597.35	18,130.92	9,610.94	7,563.65	6,868.26	3,932.12
TNM II	49,299.31	18,910.19	9,781.63	7,699.70	8,133.56	4,771.86
TNM III	49,849.37	15,523.81	10,590.69	7,759.96	7,497.57	4,327.84
TNM IV	64,724.01	19,828.96	15,430.45	8,767.87	11,112.75	6,096.37
P-value	0.113	0.910	0.151	0.098	0.008	0.166

Note: Costs are measured by 2020 CNY (¥) and are presented as medians

a Comparisons between groups are conducted using the Kruskal-Wallis Test. Results are presented as P-values

b Comparisons between groups are conducted using the Jonckheere-Terpstra Test. Results are presented as P-values

Multivariable analysis was conducted and the results, displayed in Table 4, were similar to the findings presented in Table 3: Higher age at diagnosis and more advanced tumor stage were generally associated with higher SC medical costs, particularly for patients with tumors in the middle and lower parts of the stomach. No significant difference was observed between male and female, except for medication and examination costs for middle stomach tumors.

**Table 4:** Associations between cost of stomach cancer and patient characteristics by cost category among patients with stomach cancer who underwent surgery by tumor site

Characteristics Tumor Sites	Total Cost	Material Cost	Medication Cost	Surgery Cost	Examination Cost	Hospital Service Cost
The Upper Part of Stomach						
**Sex**						
Male	Reference	Reference	Reference	Reference	Reference	Reference
Female	-0.030	0.003	-0.054	0.020	-0.042	-0.081
	(-0.14,0.08)	(-0.18,0.18)	(-0.24,0.13)	(-0.07,0.11)	(-0.18,0.10)	(-0.26,0.10)
**Age at Diagnosis**						
≤ 59	Reference	Reference	Reference	Reference	Reference	Reference
60–79	-0.032	-0.049	-0.101	-0.082	0.040	0.092
	(-0.14,0.08)	(-0.23,0.13)	(-0.24,0.04)	(-0.18,0.02)	(-0.12,0.20)	(-0.11,0.29)
≥ 80	0.037	-0.191	0.084	-0.047	0.189	0.489
	(-0.12,0.19)	(-0.44,0.06)	(-0.10,0.27)	(-0.18,0.08)	(-0.08,0.46)	(0.23,0.75)^b^
**Cancer Stage**						
TNM I	Reference	Reference	Reference	Reference	Reference	Reference
TNM II	-0.137	-0.154	-0.143	-0.071	-0.061	-0.235
	(-0.24,-0.03)^a^	(-0.30,-0.01)^a^	(-0.33,0.04)	(-0.16,0.02)	(-0.21,0.09)	(-0.41,-0.06)^a^
TNM III	-0.044	-0.196	0.024	-0.020	0.105	0.094
	(-0.17,0.08)	(-0.33,-0.06)^b^	(-0.16,0.21)	(-0.13,0.09)	(-0.07,0.28)	(-0.13,0.32)
TNM IV	-0.130	-0.059	-0.282	-0.089	-0.149	-0.185
	(-0.23,-0.03)^a^	(-0.37,0.25)	(-0.62,0.05)	(-0.18,0.00)	(-0.35,0.05)	(-0.57,0.20)
The Middle Part of Stomach						
**Sex**						
Male	Reference	Reference	Reference	Reference	Reference	Reference
Female	-0.046	-0.019	-0.097	-0.014	-0.065	-0.044
	(-0.09,0.00)	(-0.08,0.05)	(-0.17,-0.02)^a^	(-0.05,0.02)	(-0.13,-0.00)^a^	(-0.13,0.05)
**Age at Diagnosis**						
≤ 59	Reference	Reference	Reference	Reference	Reference	Reference
60–79	-0.003	-0.066	0.025	-0.004	0.091	0.021
	(-0.06,0.05)	(-0.14,0.11)	(-0.07,0.12)	(-0.05,0.04)	(0.02,0.17)^a^	(-0.08,0.12)
≥ 80	0.128	-0.068	0.279	0.052	0.293	0.254
	(0.02,0.23)^a^	(-0.19,0.06)	(0.11,0.45)^b^	(-0.02,0.12)	(0.15,0.43)^b^	(0.08,0.43)^b^
**Cancer Stage**						
TNM I	Reference	Reference	Reference	Reference	Reference	Reference
TNM II	0.043	-0.025	0.080	0.000	0.139	0.135
	(-0.03,0.11)	(-0.11,0.06)	(-0.02,0.18)	(-0.05,0.05)	(0.05,0.22)^b^	(0.02,0.25)^a^
TNM III	0.045	-0.013	0.064	0.022	0.105	0.129
	(-0.01,0.10)	(-0.08,0.06)	(-0.03,0.16)	(-0.02,0.07)	(0.03,0.18)^b^	(0.02,0.23)^a^
TNM IV	0.213	0.069	0.244	0.187	0.312	0.379
	(0.03,0.39)^a^	(-0.20,0.34)	(0.04,0.45)^a^	(0.04,0.33)^a^	(0.14,0.48)^b^	(0.13,0.63)^b^
The Lower Part of Stomach						
**Sex**						
Male	Reference	Reference	Reference	Reference	Reference	Reference
Female	-0.032	-0.043	-0.052	-0.012	-0.029	0.010
	(-0.07,0.00)	(-0.09,0.01)	(-0.11,0.01)	(-0.04,0.02)	(-0.08,0.02)	(-0.06,0.08)
**Age at Diagnosis**						
≤ 59	Reference	Reference	Reference	Reference	Reference	Reference
60–79	0.023	-0.041	0.056	0.015	0.090	0.118
	(-0.03,0.07)	(-0.10,0.02)	(-0.02,0.13)	(-0.02,0.05)	(0.04,0.14)^b^	(0.01,0.22)^a^
≥ 80	0.106	-0.085	0.177	0.069	0.288	0.372
	(0.04,0.18)^b^	(-0.17,-0.00)^a^	(0.06,0.30)^b^	(0.01,0.13)^a^	(0.19,0.39)^b^	(0.24,0.51)^b^
**Cancer Stage**						
TNM I	Reference	Reference	Reference	Reference	Reference	Reference
TNM II	0.054	0.050	0.062	0.002	0.104	0.071
	(0.01,0.10)^a^	(-0.01,0.11)	(-0.01,0.13)	(-0.03,0.03)	(0.05,0.16)^b^	(-0.01,0.15)
TNM III	0.090	0.086	0.102	0.011	0.154	0.123
	(0.05,0.13)^b^	(0.03,0.14)^b^	(0.03,0.17)^b^	(-0.02,0.05)	(0.10,0.21)^b^	(0.04,0.20)^b^
TNM IV	0.091	0.051	0.112	0.064	0.100	0.251
	(-0.01,0.19)	(-0.05,0.15)	(-0.03,0.26)	(-0.03,0.16)	(-0.06,0.26)	(0.10,0.40)^b^
Multicentric Stomach Cancer						
**Sex**						
Male	Reference	Reference	Reference	Reference	Reference	Reference
Female	-0.005	0.013	-0.059	0.051	0.019	-0.026
	(-0.10,0.10)	(-0.14,0.16)	(-0.26,0.14)	(-0.03,0.13)	(-0.17,0.20)	(-0.26,0.21)
**Age at Diagnosis**						
≤ 59	Reference	Reference	Reference	Reference	Reference	Reference
60–79	0.070	-0.049	0.066	0.320	0.214	0.166
	(-0.05,0.19)	(-0.28,0.18)	(-0.12,0.25)	(0.05,0.59)^a^	(0.02,0.40)^a^	(-0.18,0.51)
≥ 80	0.184	-0.304	0.310	0.433	0.672	0.534
	(-0.02,0.39)	(-0.61,0.00)	(-0.01,0.63)	(0.07,0.80)^a^	(0.39,0.95)^b^	(0.08,0.98)^a^
**Cancer Stage**						
TNM I	Reference	Reference	Reference	Reference	Reference	Reference
TNM II	0.009	-0.037	0.005	0.010	0.220	0.023
	(-0.10,0.12)	(-0.19,0.12)	(-0.18,0.19)	(-0.08,0.10)	(0.05,0.39)^a^	(-0.24,0.28)
TNM III	0.008	-0.126	-0.003	0.052	0.325	0.075
	(-0.14,0.16)	(-0.34,0.09)	(-0.30,0.30)	(-0.08,0.18)	(0.12,0.53)^b^	(-0.20,0.35)
TNM IV	0.248	0.038	0.323	0.254	0.546	0.395
	(0.04,0.45)^a^	(-0.27,0.35)	(0.02,0.63)^a^	(0.06,0.45)^a^	(0.27,0.82)^b^	(-0.09,0.88)

Note: Costs were measured by 2020 CNY(¥) and were logarithm transformed before analysis. Generalized linear models were estimated separately for each tumor-site group, and results were presented as regression coefficients with 95% confidence intervals. The models included sex, age group, TNM stage, and calendar year as categorical covariates. Heteroskedasticity-robust standard errors were used. The VIFs for the regressions of the upper part of stomach, the middle part of stomach, the lower part of stomach, and the multicentric stomach cancer were 1.84, 1.62, 1.69, and 2.08, respectivelya *p* < 0.05 b *p* < 0.01

The results of the sensitivity analysis, which can be found in the additional file, were similar to those reported in Table 4, suggesting the robustness of our results (see Supplementary Table 1). In addition, the analysis using more granular age categories yielded broadly similar findings. The overall patterns of higher costs among older patients and patients with more advanced tumor stage remained evident, particularly for tumors in the middle and lower parts of the stomach (see Supplementary Table 2). In the pooled analyses, tumor site was not significantly associated with total medical cost or any cost component after adjustment for sex, age, TNM stage, and calendar year, whereas associations with age and TNM stage remained evident (Supplementary Table 3).

**Fig. 2:**
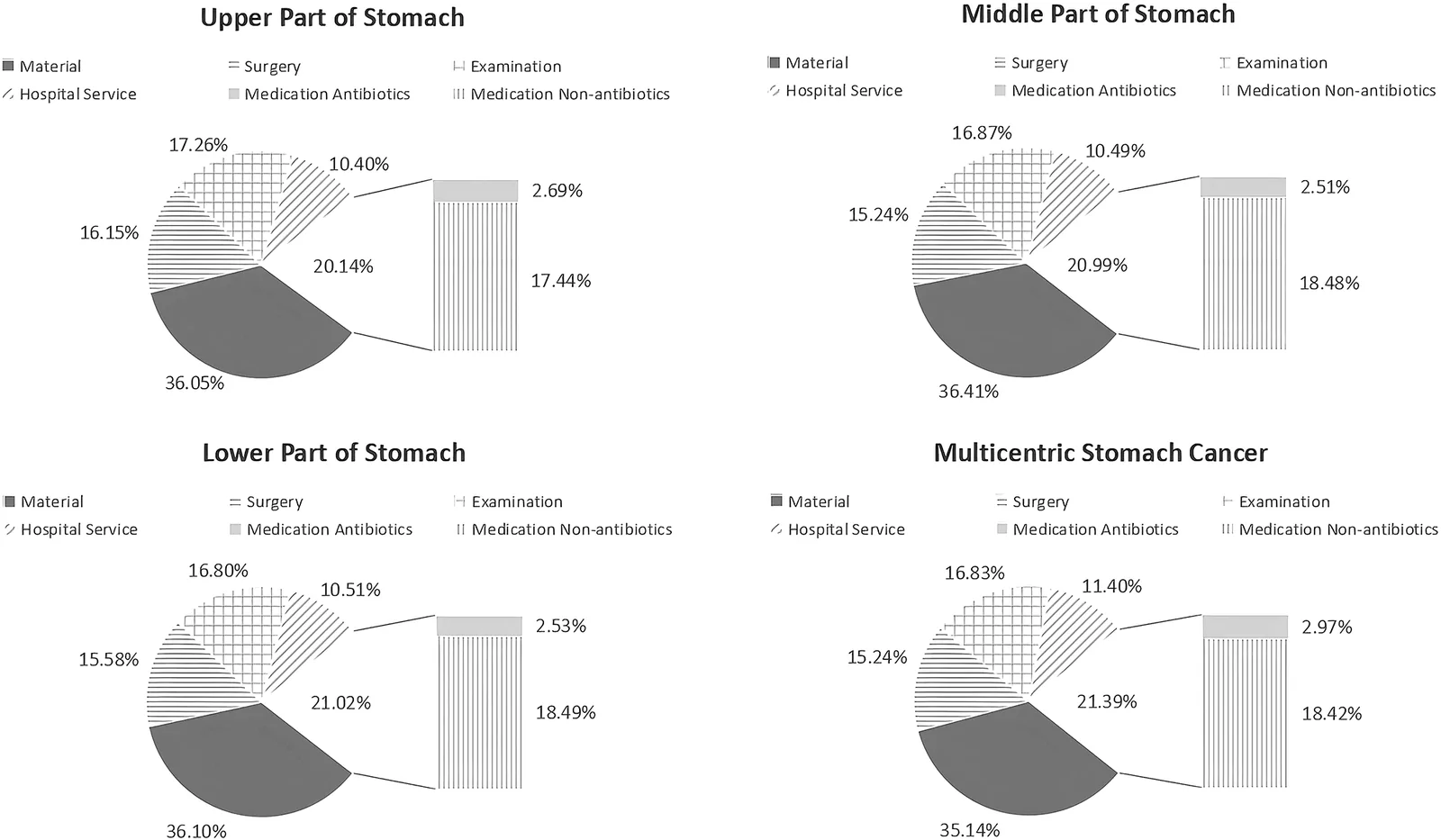
Proportional breakdown of medical costs for stomach cancer by tumor site. Note: The cost categories include material costs, medication costs (including antibiotics and non-antibiotics costs), surgery costs, examination costs, and hospital service costs

Figure 2 displays the proportional breakdown of total medical costs of patients with SC who underwent surgery by tumor site. Materials, medication, and examination were the three largest components of total costs for all tumor sites. Materials, as the major cost driver, accounted for 35–36% of total costs across tumor-site groups. Medication costs was the second largest component accounting for 20%-21% of total medical costs across tumor-site groups, followed by examination costs, which accounted for 16–17% of total costs. Medication costs were further divided into two categories: antibiotics and non-antibiotics costs, with the former accounting for only 2% of total costs and the later one accounting for 17–18%. Hospital service costs represented the smallest proportion, accounting for only 10%-12% of total costs. A similar composition of medical costs was also observed across years and tumor stages, which could be found in the additional files (see Supplementary Fig. 1 and Supplementary Fig. 2).

## Discussion

In the present study, most tumors started at the lower part of the stomach (antrum and pylorus), which was consistent with previous studies in China [20, 21], the Netherlands [22], and the USA [23]. However, SC was more likely to occur in the body of the stomach in Japan [24], Spain [25], and Switzerland [26]. The high proportion of tumors located in the lower stomach in the present study could be attributable to two reasons. First, the high rate of H. pylori infection in China may increase the risk of tumors in the lower part of the stomach, given that H. pylori infection is also a major risk factor for distal SC [27, 28]. Second, patients with SC located in the lower part may experience symptoms, such as pyloric obstruction, which could cause intractable vomiting, nausea, and poor oral intake, potentially leading to earlier detection of cancer [29].

The proportion of male patients was 2.3 times higher than that of female patients, which was similar to previous studies in China [9, 14, 15]. It was also comparable to a global study including 38 countries from Africa, Asia, Europe, the Americas, and Oceania, in which the proportion of male patients was 1.5 to 3 times higher than that of female patients [30]. This sex difference might be attributed to several reasons. First, more frequent tobacco and alcohol consumption among males might lead to a higher risk of SC [31]. Second, the predominance of SC in males might be attributed to the higher frequency of H. pylori infection in Chinese males [28], as literature demonstrated that H. pylori infection is an established risk factor for SC [32]. Third, previous studies have indicated that female patients with SC tend to have smaller tumors than male patients [33]. As a result, females may experience more subtle symptoms that may lead to delays in diagnosis. Fourth, genetic and hormonal risk factors, such as sex hormones, may contribute to the sex difference in SC prevalence. Some studies suggest that estrogen may offer some protection against SC in females [31], whereas androgen receptors may increase the SC risk in males [33]. In addition, our study showed that the sex difference in prevalence was more pronounced for patients with tumors in the upper part of the stomach (cardia and fundus), which is consistent with a recent meta-analysis [34]. This might be attributed to the suppressive impact of female sex hormones on proximal SC [35].

Interestingly, the majority of patients in our study were at stages I and II. In contrast, previous studies in China [9, 14, 15], Poland [36], and New Zealand [37] reported that most patients were at stages III and IV. However, in a Japanese study, stage I and stage II patients accounted for 70% of the total sample, which may be attributable to Japan’s national screening program for stomach cancer [38, 39]. Similarly, the larger proportion of early-stage SC patients in our study might be explained by the gastrointestinal cancer screening program implemented by the Zhejiang government in 2013 that improved the chances of early SC diagnosis [40].

The median medical treatment cost per SC inpatient between 2020 and 2023 was ¥47,539.68, whereas the average cost in a study using 2011 data was ¥43,249.00 [14]. This indicates an increasing economic burden for SC patients. Our findings, however, were lower than those of the studies using data from 2014 (¥55,463.34) [9] and 2016 (¥69,405.39) [15]. This difference might be attributed to the larger proportion of stage I patients in our study (46.28%) compared with previous studies (11.50–17.5%) [9, 15], highlighting the crucial role that early diagnosis plays in reducing the economic burden of SC. Materials, medications, and examinations were the three largest contributors to SC-related medical costs, which is comparable to previous studies in China [14, 15]. This pattern was consistent regardless of tumor site, tumor stage, and year at diagnosis. Expenses on materials accounted for 35–36% of total costs across tumor-site groups, which was higher than a Chinese study in 2016 (25.2%) [15]. The increase in the proportion of material costs might be attributable to technological advances in SC treatment over the past decade [41, 42]. In addition, the Chinese government’s efforts to regulate the cancer drug market and reform the medical insurance system, which reduced the cancer-related medication costs, might also have contributed to the lower proportion of material costs in our study [10, 11]. This can also be supported by the lower proportion of medication costs in our study (20–21%) compared with studies using data from 2011 (53.00%) [14] and 2016 (42.70%) [15]. The proportion of medication costs in our study was much lower than reported in some foreign studies in Turkey (36.83%) [43], Iran (35.32%) [44], and the USA (59.57%) [45], but higher than in South Korea (1.6%) [46]. Such disparities might be explained by the differences in health care systems and drug market regulations across countries. Regarding the composition of medication costs, antibiotics costs only accounted for around 2% of total costs (i.e., 11–13% of medication costs) across tumor-site groups. This might reflect the Chinese government’s efforts to regulate the misuse and overuse of antibiotics in medical practice since 2016 [47]. Examination costs accounted for 16%-17% of total medical treatment costs, which was comparable to a study in Iran (21.14%) [44], but higher than those reported in Turkey (6.01%) [43] and South Korea (11.27%) [46].

Surgery costs ranked the fourth largest cost driver, accounting for15-16% of total medical costs. This proportion was like that in South Korea (16.80%) [46] but higher than that in Iran (9.50%) [44]. A potential explanation for this disparity might be differences in preferred SC surgery strategies across countries: total gastrectomy was more commonly adopted in Iran than in China and Korea [48, 49]. Total gastrectomy may lead to complications and prolonged hospitalization, which could increase medical treatment costs [50]. As a result, the proportion of the surgery costs in China and South Korea might be higher because the lower prevalence of total gastrectomy could reduce the overall medical costs. Another possible explanation is East Asia’s advantage in the developing and adopting advanced SC surgical techniques, such as minimally invasive techniques [51]. These techniques can lead to overall cost savings due to rapid recovery and reduced hospital stay, and therefore may increase the proportion of surgery costs relative to other cost drivers [52].

Notably, the proportions of material and medication costs in 2023 had declined compared with 2020 for all tumor sites except multicentric SC. The decline in the proportions of medication and material costs in total costs could be attributable to the Chinese government’s efforts to regulate the medical market and reform the public medical insurance system to relieve the economic burden of SC patients [10, 11]. However, for multicentric SC, the proportions of all cost categories increased from 2020 to 2023. This might be because treating multicentric SC requires extensive interventions and more complicated therapies, which may have contributed to higher overall medical costs [19]. The proportion of the hospital service costs in multicentric SC declined substantially from 2020 to 2023, mainly due to a sudden drop in 2021. The drop in 2021 might be attributed to the limited sample size that was more vulnerable to the influence of extreme values.

Medical costs for SC were also found to vary across patients’ sociodemographic and clinical characteristics. SC medical costs generally increased with the age at diagnosis, consistent with previous literature [14, 15]. This finding was expected, as older patients may incur additional expenses for treating complications and comorbidities. In addition, in the present study, SC medical costs increased with the tumor stage, which was consistent with some studies [9, 14] but different from a study that reported decreasing medical costs with tumor stage [15]. The divergent patterns may be explained by the disparities in treatment strategy selection, as patients with later-stage disease may choose between intensive and conservative treatments, leading to different trends in costs. No significant difference was observed in the cost of SC treatment between male and female patients, except for medication and examination costs for middle stomach tumors. Male patients with middle stomach tumors had significantly higher medication and examination costs than female patients. A possible explanation is that male patients may generally have a greater burden of comorbidities in related to SC, such as cardiovascular disease, cerebral disease, and renal disease [53]. These comorbidities may require more pre-operative tests and examinations, as well as additional supportive drugs to manage side effects of SC treatment, leading to higher costs in examination and medication. Although a significant sex difference in SC costs was found only in the middle stomach cancer group, this does not necessarily mean that sex does not matter for other tumor sites. Tumors in the upper and lower parts of the stomach are usually accompanied by more distinct symptoms, whereas tumors in the body of the stomach may cause vague symptoms. For example, cardia cancer may present with gastroesophageal reflux disease [54], and pylorus cancer may be accompanied with nausea and emesis [55]. These distinct features may mask the effect of sex different in influencing treatment costs.

The findings have important implications for hospital resource planning, cost management, and healthcare payment policy for patients with SC who underwent surgery, particularly in settings with patient population structures and treatment practices similar to those of the study hospital. Materials, medications, and examinations accounted for the majority of total medical costs, and therefore may warrant particular attention in healthcare reimbursement policy, procurement planning, and cost monitoring. Policymakers may consider reviewing the reimbursement coverage for clinically necessary items in these categories and enhancing financial protection for patients, particularly those who are economically vulnerable or require high-cost treatment. In particular, improving reimbursement for clinically necessary high-cost medications, examinations, and materials may contribute to stronger financial protection, especially among patients with limited ability to pay. In addition, older age and more advanced cancer stage were associated with higher costs, particularly among patients with tumors in the middle and lower parts of the stomach. These findings may help hospitals better allocate resources across different patient groups and provide tailored treatment strategies. Besides, SC prevention programs have been government-subsidized in Japan and South Korea [56]. A screening program aimed at promoting the early diagnosis of SC among old people had been implemented in Zhejiang Province with the support of the provincial government, but it has not been implemented as a national program in China. The Chinese government could consider subsidizing various SC prevention programs, such as population-based SC screening or eradication of H. pylori infection, to help reduce the SC-related economic burden.

The present study has several strengths. First, SC-related medical costs were analyzed by separating them into five subcategories using recent data extracted from real-life clinical settings. Understanding the components of SC costs is crucial for effective management of treatment and allocation of medical resources for patients with SC who underwent surgery. Previous studies either did not provide a detailed breakdown of medical costs [9, 12, 13] or relied on data from the 2000s and 2010s, which may not fully reflect contemporary treatment practices, prices, procurement arrangements, and healthcare payment policies [14, 15]. Second, this study integrated tumor-site stratification within a detailed cost-composition framework. Based on real-life hospitalization records, we not only estimated the SC-related medical costs, but also analyzed the proportional breakdown of costs for patients with tumors located in different parts of the stomach. This allows a deeper understanding of the heterogeneity in the medical costs caused by different growth patterns and symptoms in relation to different tumor sites, whereas this clinical heterogeneity is often ignored in previous studies that used only aggregated SC data [9, 12–15]. Third, all costs were adjusted to 2020 Chinese yuan, and multivariable and sensitivity analyses were conducted to assess the consistency of the findings. These estimates may support hospital resource planning and provide cost inputs for future economic evaluations of SC prevention and treatment strategies.

However, several limitations should also be noted. First, this study only considered inpatient costs of SC treatment. We did not consider outpatient costs (e.g. radiotherapy and chemotherapy), ICU costs, or indirect costs (e.g. transportation, hired nursing, additional nutrition), which may have led to underestimation of the overall SC-related costs. Second, this study did not include information on patients’ complications and comorbidities, which may have missed some important insights into the cost drivers of SC-related medical costs. Third, this study was a single-center study conducted at one tertiary hospital in Ningbo, which may limit the representativeness of the study population and the generalizability of our findings to other hospitals and regions. In addition, SC in China exhibits significant regional heterogeneity in incidence and mortality rates [57], which may affect local SC medical costs and further limit the generalizability of our findings. Fourth, we only collected data from the study hospital where the patients were diagnosed and hospitalized, which did not include expenses incurred at other hospitals, as some patients may have elected hospital transfer. Besides, our study focused on hospitalized patients with SC who underwent surgery. In clinical practice, some later-stage patients (stages III and IV) or medically inoperable patients may not be elected for surgical treatment and thus were not included in this study. This may affect our estimates of SC-related medical costs. Fifth, although the pooled GLM analysis was conducted to assess the independent association between tumor site and SC-related medical costs after adjustment for covariates, some tumor-site subgroups still had relatively small sample sizes, particularly the upper part subgroup (*n* = 80) and the multicentric SC subgroup (*n* = 63). These small subgroup sample sizes may reduce the statistical power of the stratified analyses and result in less precise and less stable regression estimates. In addition, estimates in smaller subgroups may be more sensitive to extreme values. Therefore, the subgroup regression results should be interpreted with caution, and insignificant associations observed in these subgroups should not necessarily be interpreted as evidence of no true association. Accordingly, future researchers may conduct multi-center studies that incorporate larger samples, a broader range of costs, and more detailed clinical information for comparative analyses to enhance our understanding of the economic burden of SC. Another valuable direction for future research is to further examine whether the associations between patients’ sociodemographic and clinicopathological characteristics and SC-related medical costs differ across other subgroups, such as sex, age, and TNM stage, to provide a more detailed understanding of cost patterns among different patient populations.

## Conclusions

This hospital-based retrospective study estimated the inpatient medical costs and their composition among patients with SC who underwent surgery across different tumor sites in real-life clinical settings in Ningbo, China. Materials, medications, and examinations accounted for the largest proportions of SC treatment costs. Medical costs generally increased with the age at diagnosis and tumor stage. This pattern was typically observed for patients with tumors in the body and lower parts of the stomach. These findings have important implications for hospital resource planning, cost management, and healthcare payment policy for SC patients. Identification of major cost components of SC may help optimize procurement and reimbursement strategies for SC hospitalization. The associations between higher costs with advanced tumor stage and older age may provide insights for SC prevention and treatment strategies. Future research should incorporate outpatient and indirect costs and data from other regions of China to provide a more comprehensive evaluation of the economic burden of SC.

## Supplementary Information

Below is the link to the electronic supplementary material.

Supplementary Material 1: Additional file 1 in PDF: Supplementary Figure 1. Proportional Breakdown of Medical Costs for Stomach Cancer by Tumor Stage (I, II, III, IV).

Supplementary Material 2: Additional file 2 in PDF: Supplementary Figure 2. Proportional Breakdown of Medical Costs for Stomach Cancer by Year.

Supplementary Material 3: Additional file 3 in docx: Supplementary Table 1. Associations between Cost of Stomach Cancer and Patient Characteristics by Cost Category among Patients with Stomach Cancer Who Underwent Surgery by Tumor Site after Winsorization.

Supplementary Material 4: Additional file 4 in docx: Supplementary Table 2. Associations between Cost of Stomach Cancer and Patient Characteristics by Cost Category among Patients with Stomach Cancer Who Underwent Surgery by Tumor Site Using More Granular Age Categories.

Supplementary Material 5: Additional file 5 in docx: Supplementary Table 3. Associations between Cost of Stomach Cancer and Patient Characteristics by Cost Category among Patients with Stomach Cancer Who Underwent Surgery.

## Data Availability

The data are not publicly available due to privacy and ethical restrictions. De-identified data may be obtained from the corresponding authors upon reasonable request, subject to institutional and ethical approvals. The Stata do-files used for the GLM and additional analyses, including the sensitivity analyses, are also available from the corresponding authors upon reasonable request.

## Abbreviations

CPI: Consumer Price Index
CNY: Chinese Yuan
DRG: Diagnosis-Related Group
GLM: Generalized Linear Model
OTC: Over-the-Counter
SC: Stomach Cancer
TNM: Tumor, Nodes, Metastasis
USA: United States of America
