# Supplementary material for: Inpatient medical costs and their composition among patients with stomach cancer who underwent surgery by tumor location: a hospital-based retrospective study in China
Source: Cost Eff Resour Alloc. 2026 Oct 1;24:122. doi: 10.1186/s12962-026-00837-7 (PMC13637226; doi:10.1186/s12962-026-00837-7)
Supplement: Supplementary file 1 [file 12962_2026_837_MOESM1_ESM.pdf]

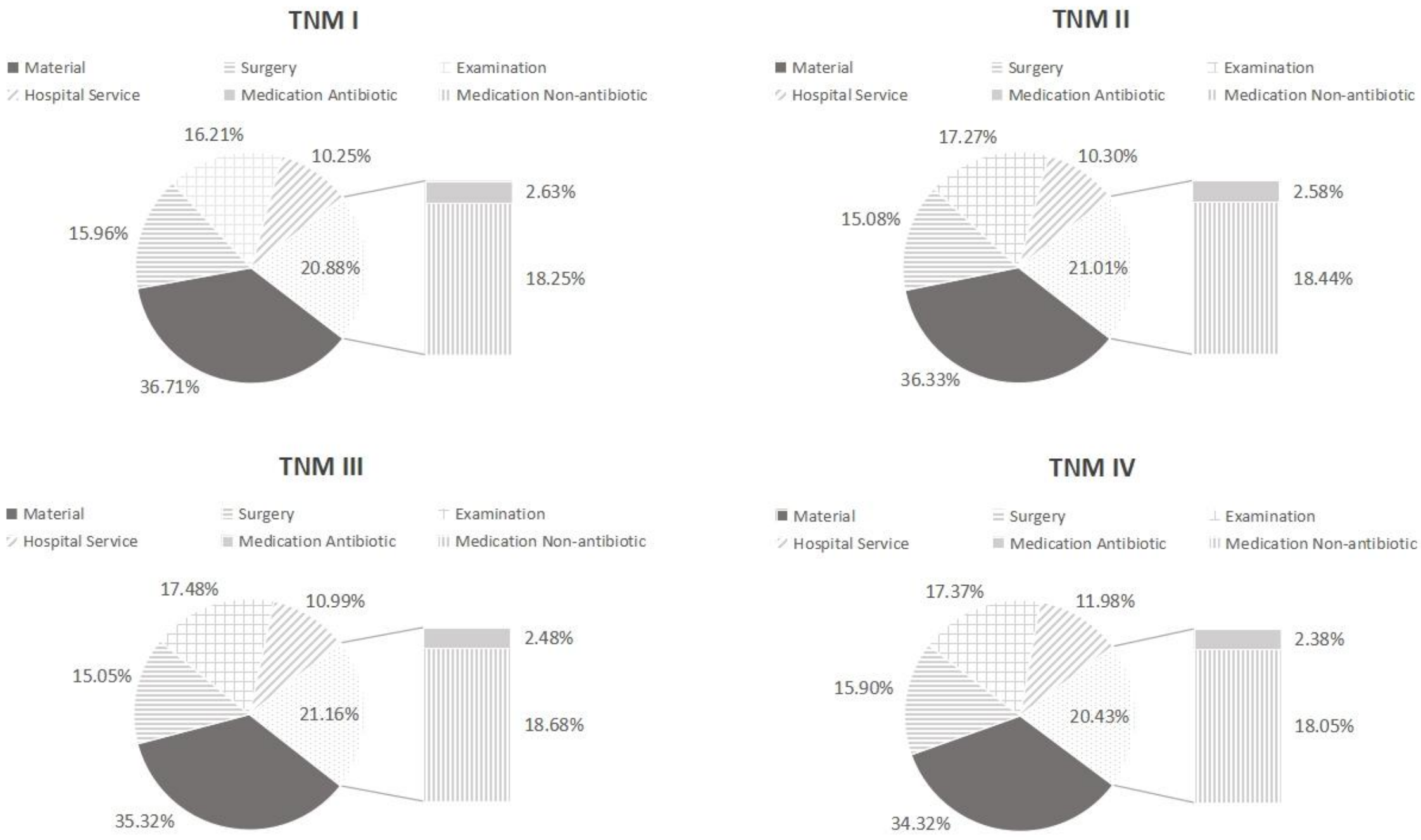

Supplementary Figure 1. Proportional Breakdown of Medical Costs for Stomach Cancer by Tumor Stage (I, II, III, IV).

Note: The cost categories include material costs, medication costs (including antibiotics and non-antibiotics costs), surgery costs, examination costs, and hospital service costs.
