# Supplementary material for: Inpatient medical costs and their composition among patients with stomach cancer who underwent surgery by tumor location: a hospital-based retrospective study in China
Source: Cost Eff Resour Alloc. 2026 Oct 1;24:122. doi: 10.1186/s12962-026-00837-7 (PMC13637226; doi:10.1186/s12962-026-00837-7)
Supplement: Supplementary file 4 [file 12962_2026_837_MOESM4_ESM.docx]

**Supplementary Table 2.** Associations between Cost of Stomach Cancer and Patient Characteristics by Cost Category among Patients with Stomach Cancer Who Underwent Surgery by Tumor Site Using More Granular Age Categories

| **Characteristics**  **Tumor Sites** | **Total Cost** | **Material Cost** | **Medication Cost** | **Surgery Cost** | **Examination Cost** | **Hospital Service Cost** |
| --- | --- | --- | --- | --- | --- | --- |
|  |  |  |  |  |  |  |
| The Upper Part of Stomach | | | | | | |
| **Sex** |  |  |  |  |  |  |
| Male | Reference | Reference | Reference | Reference | Reference | Reference |
| Female | -0.040 | 0.003 | -0.078 | 0.018 | -0.046 | -0.117 |
|  | (-0.16,0.08) | (-0.18,0.18) | (-0.26,0.11) | (-0.08,0.11) | (-0.20,0.11) | (-0.32,0.09) |
| **Age at Diagnosis** |  |  |  |  |  |  |
| ≤59 | Reference | Reference | Reference | Reference | Reference | Reference |
| 60-64 | -0.041 | -0.026 | -0.110 | -0.051 | -0.025 | -0.046 |
|  | (-0.17,0.08) | (-0.23,0.18) | (-0.30,0.08) | (-0.19,0.09) | (-0.27,0.22) | (-0.29,0.20) |
| 65-69 | -0.063 | -0.052 | -0.099 | -0.084 | -0.095 | 0.072 |
|  | (-0.22,0.09) | (-0.27,0.17) | (-0.33,0.13) | (-0.24,0.07) | (-0.30,0.11) | (-0.22,0.36) |
| 70-74 | 0.014 | -0.048 | -0.024 | -0.074 | 0.120 | 0.230 |
|  | (-0.12,0.15) | (-0.26,0.16) | (-0.20,0.15) | (-0.20,0.05) | (-0.07,0.31) | (-0.01,0.47) |
| 75-79 | -0.076 | -0.081 | -0.220 | -0.136 | 0.096 | 0.066 |
|  | (-0.22,0.06) | (-0.31,0.14) | (-0.44,-0.00)^a^ | (-0.24,-0.03)^a^ | (-0.12,0.31) | (-0.20,0.33) |
| ≥80 | 0.035 | -0.194 | 0.081 | -0.052 | 0.190 | 0.503 |
|  | (-0.12,0.19) | (-0.44,0.06) | (-0.10,0.26) | (-0.18,0.08) | (-0.08,0.46) | (0.25,0.76)^b^ |
| **Cancer Stage** |  |  |  |  |  |  |
| TNM I | Reference | Reference | Reference | Reference | Reference | Reference |
| TNM II | -0.118 | -0.147 | -0.105 | -0.058 | -0.050 | -0.202 |
|  | (-0.22,-0.02)^a^ | (-0.30,0.00) | (-0.27,0.06) | (-0.15,0.03) | (-0.20,0.10) | (-0.37,-0.03)^a^ |
| TNM III | -0.031 | -0.192 | 0.044 | -0.013 | 0.125 | 0.109 |
|  | (-0.14,0.08) | (-0.33,-0.06)^b^ | (-0.13,0.22) | (-0.11,0.09) | (-0.05,0.31) | (-0.10,0.32) |
| TNM IV | -0.118 | -0.070 | -0.257 | -0.101 | -0.118 | -0.086 |
|  | (-0.24,0.00) | (-0.37,0.23) | (-0.60,0.08) | (-0.22,0.08) | (-0.35,0.11) | (-0.46,0.29) |
| The Middle Part of Stomach | | | | | | |
| **Sex** |  |  |  |  |  |  |
| Male | Reference | Reference | Reference | Reference | Reference | Reference |
| Female | -0.046 | -0.021 | -0.095 | -0.013 | -0.063 | -0.042 |
|  | (-0.09,0.00) | (-0.09,0.04) | (-0.17,-0.02)^a^ | (-0.05,0.02) | (-0.13,0.00) | (-0.13,0.05) |
| **Age at Diagnosis** |  |  |  |  |  |  |
| ≤59 | Reference | Reference | Reference | Reference | Reference | Reference |
| 60-64 | -0.023 | -0.082 | 0.023 | -0.022 | 0.049 | -0.014 |
|  | (-0.11,0.07) | (-0.19,0.03) | (-0.13,0.18) | (-0.08,0.04) | (-0.06,0.16) | (-0.19,0.16) |
| 65-69 | -0.024 | -0.049 | -0.024 | -0.025 | 0.032 | -0.029 |
|  | (-0.09,0.05) | (-0.15,0.05) | (-0.14,0.09) | (-0.08,0.03) | (-0.06,0.12) | (-0.16,0.10) |
| 70-74 | 0.002 | -0.100 | 0.048 | 0.007 | 0.137 | 0.053 |
|  | (-0.07,0.08) | (-0.19,-0.01)^a^ | (-0.07,0.17) | (-0.05,0.07) | (0.05,0.23)^b^ | (-0.08,0.18) |
| 75-79 | 0.037 | -0.023 | 0.060 | 0.023 | 0.133 | 0.067 |
|  | (-0.05,0.12) | (-0.15,0.10) | (-0.07,0.19) | (-0.05,0.09) | (0.02,0.25)^a^ | (-0.06,0.19) |
| ≥80 | 0.128 | -0.069 | 0.280 | 0.052 | 0.293 | 0.255 |
|  | (0.02,0.23)^a^ | (-0.19,0.06) | (0.11,0.45)^b^ | (-0.02,0.12) | (0.15,0.43)^b^ | (0.08,0.43)^b^ |
| **Cancer Stage** |  |  |  |  |  |  |
| TNM I | Reference | Reference | Reference | Reference | Reference | Reference |
| TNM II | 0.042 | -0.025 | 0.078 | -0.002 | 0.135 | 0.132 |
|  | (-0.03,0.11) | (-0.11,0.06) | (-0.03,0.18) | (-0.05,0.05) | (0.05,0.22)^b^ | (0.02,0.25)^a^ |
| TNM III | 0.049 | -0.013 | 0.068 | 0.026 | 0.114 | 0.136 |
|  | (-0.01,0.11) | (-0.08,0.06) | (-0.03,0.16) | (-0.02,0.07) | (0.04,0.19)^b^ | (0.03,0.24)^a^ |
| TNM IV | 0.212 | 0.072 | 0.243 | 0.185 | 0.305 | 0.374 |
|  | (0.03,0.40)^a^ | (-0.21,0.35) | (0.04,0.45)^a^ | (0.04,0.33)^a^ | (0.14,0.48)^b^ | (0.13,0.62)^b^ |
| The Lower Part of Stomach | | | | | | |
| **Sex** |  |  |  |  |  |  |
| Male | Reference | Reference | Reference | Reference | Reference | Reference |
| Female | -0.032 | -0.039 | -0.054 | -0.011 | -0.033 | 0.008 |
|  | (-0.07,0.00) | (-0.09,0.01) | (-0.11,0.00) | (-0.04,0.02) | (-0.08,0.01) | (-0.06,0.07) |
| **Age at Diagnosis** |  |  |  |  |  |  |
| ≤59 | Reference | Reference | Reference | Reference | Reference | Reference |
| 60-64 | -0.024 | -0.062 | -0.024 | 0.003 | 0.020 | -0.001 |
|  | (-0.08,0.04) | (-0.15,0.09) | (-0.12,0.07) | (-0.04,0.04) | (-0.06,0.10) | (-0.12,0.12) |
| 65-69 | 0.019 | -0.002 | 0.025 | 0.017 | 0.055 | 0.099 |
|  | (-0.04,0.08) | (-0.08,0.08) | (-0.07,0.12) | (-0.02,0.05) | (-0.01, 0.12) | (-0.02,0.22) |
| 70-74 | 0.023 | -0.051 | 0.066 | 0.019 | 0.096 | 0.122 |
|  | (-0.03,0.08) | (-0.12,0.02) | (-0.02,0.16) | (-0.02,0.06) | (0.03,0.16)^b^ | (0.01,0.24)^a^ |
| 75-79 | 0.063 | -0.065 | 0.144 | 0.015 | 0.184 | 0.231 |
|  | (-0.00,0.13) | (-0.15,0.02) | (0.04,0.25)^b^ | (-0.03,0.06) | (0.11,0.26)^b^ | (0.11,0.36)^b^ |
| ≥80 | 0.106 | -0.086 | 0.177 | 0.068 | 0.289 | 0.373 |
|  | (0.04,0.18)^b^ | (-0.17,-0.00)^a^ | (0.06,0.30)^b^ | (0.01,0.13)^a^ | (0.19,0.39)^b^ | (0.24,0.51)^b^ |
| **Cancer Stage** |  |  |  |  |  |  |
| TNM I | Reference | Reference | Reference | Reference | Reference | Reference |
| TNM II | 0.053 | 0.050 | 0.061 | 0.003 | 0.102 | 0.069 |
|  | (0.01,0.10)^a^ | (-0.01,0.11) | (-0.01,0.13) | (-0.03,0.03) | (0.05,0.16)^b^ | (-0.01,0.15) |
| TNM III | 0.090 | 0.089 | 0.102 | 0.012 | 0.153 | 0.124 |
|  | (0.05,0.13)^b^ | (0.04,0.14)^b^ | (0.03,0.17)^b^ | (-0.02,0.05) | (0.10,0.21)^b^ | (0.04,0.20)^b^ |
| TNM IV | 0.094 | 0.053 | 0.118 | 0.064 | 0.106 | 0.260 |
|  | (-0.01,0.20) | (-0.05,0.15) | (-0.04,0.27) | (-0.03,0.16) | (-0.06,0.28) | (0.10,0.42)^b^ |
| Multicentric Stomach Cancer | | | | | | |
| **Sex** |  |  |  |  |  |  |
| Male | Reference | Reference | Reference | Reference | Reference | Reference |
| Female | -0.037 | -0.013 | -0.067 | 0.055 | -0.041 | -0.095 |
|  | (-0.14,0.06) | (-0.16,0.13) | (-0.27,0.14) | (-0.02,0.13) | (-0.21,0.13) | (-0.37,0.18) |
| **Age at Diagnosis** |  |  |  |  |  |  |
| ≤59 | Reference | Reference | Reference | Reference | Reference | Reference |
| 60-64 | 0.149 | 0.019 | 0.079 | 0.308 | 0.360 | 0.337 |
|  | (-0.00,0.30) | (-0.23,0.27) | (-0.17,0.33) | (0.04,0.58)^a^ | (0.13,0.59)^b^ | (-0.07,0.74) |
| 65-69 | 0.009 | -0.082 | 0.033 | 0.311 | 0.102 | 0.051 |
|  | (-0.12,0.13) | (-0.34,0.17) | (-0.16,0.23) | (0.04,0.59)^a^ | (-0.10,0.30) | (-0.34,0.44) |
| 70-74 | 0.012 | -0.087 | 0.000 | 0.324 | 0.118 | 0.099 |
|  | (-0.15,0.17) | (-0.35,0.18) | (-0.29,0.29) | (0.05,0.60)^a^ | (-0.13,0.37) | (-0.33,0.53) |
| 75-79 | 0.171 | -0.061 | 0.309 | 0.389 | 0.379 | 0.193 |
|  | (-0.00,0.35) | (-0.36,0.24) | (-0.03,0.64) | (0.08,0.69)^a^ | (0.09,0.67)^b^ | (-0.25,0.64) |
| ≥80 | 0.170 | -0.319 | 0.318 | 0.438 | 0.647 | 0.497 |
|  | (-0.04,0.38) | (-0.62,-0.01)^a^ | (-0.02,0.65) | (0.07,0.81)^a^ | (0.36,0.93)^b^ | (0.04,0.96)^a^ |
| **Cancer Stage** |  |  |  |  |  |  |
| TNM I | Reference | Reference | Reference | Reference | Reference | Reference |
| TNM II | 0.018 | -0.027 | -0.008 | 0.008 | 0.240 | 0.058 |
|  | (-0.08,0.12) | (-0.19,0.14) | (-0.18,0.16) | (-0.09,0.10) | (0.08,0.40)^b^ | (-0.19,0.31) |
| TNM III | 0.045 | -0.107 | 0.043 | 0.056 | 0.388 | 0.118 |
|  | (-0.09,0.18) | (-0.31,0.10) | (-0.24,0.33) | (-0.07,0.19) | (0.17,0.61)^b^ | (-0.16,0.40) |
| TNM IV | 0.270 | 0.049 | 0.339 | 0.258 | 0.586 | 0.434 |
|  | (0.07,0.47)^b^ | (-0.26,0.36) | (0.03,0.65)^a^ | (0.06,0.46)^a^ | (0.31,0.87)^b^ | (-0.04,0.91) |
| **Note:** Costs were measured by 2020 CNY(¥) and were logarithm transformed before analysis.  Generalized linear models were estimated separately for each tumor-site group, and results were presented as regression coefficients with 95% confidence intervals.  The models included sex, age group, TNM stage, and calendar year as categorical covariates. Heteroskedasticity-robust standard errors were used. The VIFs for the regressions of the upper part of stomach, the middle part of stomach, the lower part of stomach, and the multicentric stomach cancer were 1.71, 1.48, 1.53, and 1.93, respectively. a p<0.05 b p<0.01 | | | | | | |
