# Supplementary material for: Inpatient medical costs and their composition among patients with stomach cancer who underwent surgery by tumor location: a hospital-based retrospective study in China
Source: Cost Eff Resour Alloc. 2026 Oct 1;24:122. doi: 10.1186/s12962-026-00837-7 (PMC13637226; doi:10.1186/s12962-026-00837-7)
Supplement: Supplementary file 5 [file 12962_2026_837_MOESM5_ESM.docx]

**Supplementary Table 3.** Associations between Cost of Stomach Cancer and Patient Characteristics by Cost Category among Patients with Stomach Cancer Who Underwent Surgery

| **Characteristics**  **Tumor Sites** | **Total Cost** | **Material Cost** | **Medication Cost** | **Surgery Cost** | **Examination Cost** | **Hospital Service Cost** |
| --- | --- | --- | --- | --- | --- | --- |
|  |  |  |  |  |  |  |
| **Sex** |  |  |  |  |  |  |
| Male | Reference | Reference | Reference | Reference | Reference | Reference |
| Female | -0.037 | -0.033 | -0.070 | -0.042 | -0.009 | -0.009 |
|  | (-0.06,-0.01)^b^ | (-0.07,0.00) | (-0.12,-0.03)^b^ | (-0.08-0.01)^a^ | (-0.03,0.01) | (-0.06,0.04) |
| **Age at Diagnosis** |  |  |  |  |  |  |
| ≤59 | Reference | Reference | Reference | Reference | Reference | Reference |
| 60-79 | 0.017 | -0.051 | 0.044 | 0.100 | 0.016 | 0.099 |
|  | (-0.02,0.05) | (-0.10,-0.01)^a^ | (-0.01,0.10) | (0.06,0.14)^b^ | (-0.01,0.04) | (0.03,0.17)^b^ |
| ≥80 | 0.118 | -0.096 | 0.222 | 0.310 | 0.072 | 0.362 |
|  | (0.06,0.17)^b^ | (-0.16,-0.03)^b^ | (0.13,0.31)^b^ | (0.24,0.38)^b^ | (0.03,0.12)^b^ | (0.26,0.46)^b^ |
| **Tumor Site** |  |  |  |  |  |  |
| Upper Part | Reference | Reference | Reference | Reference | Reference | Reference |
| Middle Part | 0.028 | 0.025 | 0.059 | 0.022 | -0.011 | 0.046 |
|  | (-0.03,0.08) | (-0.04,0.09) | (-0.02,0.14) | (-0.05,0.10) | (-0.06,0.04) | (-0.05,0.14) |
| Lower Part | -0.008 | -0.017 | 0.030 | -0.023 | -0.037 | 0.005 |
|  | (-0.06,0.04) | (-0.08,0.05) | (-0.05,0.11) | (-0.10, 0.05) | (-0.08,0.01) | (-0.09,0.10) |
| Multicentric | 0.030 | 0.005 | 0.091 | -0.020 | -0.036 | 0.072 |
|  | (-0.04,0.10) | (-0.09,0.10) | (-0.02,0.20) | (-0.12,0.08) | (-0.10,0.03) | (-0.07,0.21) |
| **Cancer Stage** |  |  |  |  |  |  |
| TNM I | Reference | Reference | Reference | Reference | Reference | Reference |
| TNM II | 0.034 | 0.014 | 0.045 | 0.106 | -0.003 | 0.058 |
|  | (0.00,0.07)^a^ | (-0.03,0.06) | (-0.01,0.10) | (0.06,0.15)^b^ | (-0.03,0.02) | (-0.00 - 0.12) |
| TNM III | 0.060 | 0.030 | 0.077 | 0.135 | 0.012 | 0.114 |
|  | (0.03,0.09)^b^ | (-0.01,0.07) | (0.02,0.13)^b^ | (0.09,0.18)^b^ | (-0.01,0.04) | (0.05,0.18)^b^ |
| TNM IV | 0.126 | 0.047 | 0.143 | 0.181 | 0.107 | 0.269 |
|  | (0.04,0.21)^b^ | (-0.06,0.16) | (0.03,0.25)^a^ | (0.07,0.29)^b^ | (0.04,0.18)^b^ | (0.14,0.40)^b^ |
| **Note:** Costs were measured by 2020 CNY(¥) and were logarithm transformed before analysis.  Generalized linear models were estimated and results were presented as regression coefficients with 95% confidence intervals.  The models included sex, age group, TNM stage, tumor site, and calendar year as categorical covariates. Heteroskedasticity-robust standard errors were used. The VIF for the regressions was 2.18. a p<0.05 b p<0.01 | | | | | | |
